# Embryonic Stem Cells Exhibit mRNA Isoform Specific Translational Regulation

**DOI:** 10.1371/journal.pone.0143235

**Published:** 2016-01-22

**Authors:** Queenie Wing-Lei Wong, Candida Vaz, Qian Yi Lee, Tian Yun Zhao, Raymond Luo, Stuart K. Archer, Thomas Preiss, Vivek Tanavde, Leah A. Vardy

**Affiliations:** 1 Institute of Medical Biology, A*STAR, 8A Biomedical Grove, Immunos, 138648, Singapore, Singapore; 2 Bioinformatics Institute, A*STAR, 30 Biopolis Street, 138671, Singapore, Singapore; 3 Life Technologies, 10 Biopolis Road, 138670, Singapore, Singapore; 4 Monash Bioinformatics Platform, Monash University, Clayton, Victoria, Australia; 5 EMBL–Australia Collaborating Group, Department of Genome Science, The John Curtin School of Medical Research (JCSMR), The Australian National University, Acton (Canberra), Australian Capital Territory, Australia; 6 Victor Chang Cardiac Research Institute, Darlinghurst (Sydney), New South Wales, Australia; 7 School of Biological Sciences, Nanyang Technological University, 60 Nanyang Drive, 637551, Singapore, Singapore; University of British Columbia, CANADA

## Abstract

The presence of multiple variants for many mRNAs is a major contributor to protein diversity. The processing of these variants is tightly controlled in a cell-type specific manner and has a significant impact on gene expression control. Here we investigate the differential translation rates of individual mRNA variants in embryonic stem cells (ESCs) and in ESC derived Neural Precursor Cells (NPCs) using polysome profiling coupled to RNA sequencing. We show that there are a significant number of detectable mRNA variants in ESCs and NPCs and that many of them show variant specific translation rates. This is correlated with differences in the UTRs of the variants with the 5’UTR playing a predominant role. We suggest that mRNA variants that contain alternate UTRs are under different post-transcriptional controls. This is likely due to the presence or absence of miRNA and protein binding sites that regulate translation rate. This highlights the importance of addressing translation rate when using mRNA levels as a read out of protein abundance. Additional analysis shows that many annotated non-coding mRNAs are present on the polysome fractions in ESCs and NPCs. We believe that the use of polysome fractionation coupled to RNA sequencing is a useful method for analysis of the translation state of many different RNAs in the cell.

## Introduction

Embryonic stem cells (ESCs) possess the unique ability to self-renew and differentiate into all the cells of the body. Tight regulation of gene expression is essential for cells to maintain their self-renewal state. Upon differentiation, specific cascades of gene expression changes are implemented resulting in conversion of the cells to specific cell types. These gene expression changes are regulated and coordinated on multiple levels including transcription, RNA stability, translational control as well as protein function and degradation. This ensures a tight control over protein expression which is required for self-renewal or differentiation. Changes in transcriptional control are a major driving force for differentiation and much work has focused on mapping the binding sites of transcription factors in ESCs.[1, 2] More recently, post-transcriptional control has been shown to play a major role in ESC function. A number of miRNAs are known to promote ESC self-renewal while others are important for directed differentiation. [3–5]

It is now well established that a single gene can give rise to many different mRNA variants, the majority of which will have different protein products. These variants can arise through a number of mechanisms including alternate transcription start sites (TSS), alternative splicing and alternative poly(A) tail site selection. Alternate TSSs are a major contributor to 5’UTR diversity between transcripts from the same gene. In addition there is a large amount of tissue specific usage of alternative transcription start sites and a number of examples have been shown of TSS selection being developmentally regulated [6, 7]. Alternate TSSs can lead to altered 5’UTR sequences and different start codons resulting in alterations or truncations in the N terminus of the protein. Inappropriate usage of TSSs has been associated with cancer progression highlighting the importance of tight control over TSS usage [8].

Splicing is the most significant contributor to transcript diversity and it is estimated that over 95% of genes produce alternatively spliced mRNAs. Splicing is highly regulated and many tissues show very specific splicing patterns with some genes producing up to 25 different spliced isoforms in different tissues. Advances in sequencing technologies have confirmed the widespread prevalence of alternative splicing in different tissues yet the role of the majority of variants is still unknown [9–11]. Alternative splicing is regulated by specific RNA binding proteins that bind to the pre-RNA to control the function of the splicing machinery [4]. A number of ESC specific splicing events have been characterised demonstrating the importance of splicing control in ESC maintenance [12–16].

Variant mRNA isoforms can arise from utilisation of alternative polyadenylation (APA) sites which affect the length of the 3’end of the RNA. The majority of APA sites are present in the 3’UTR so it affects 3’UTR length. [6, 17] It has been estimated that approximately 70% of genes have APA sites with each gene having an average of two. [6, 18] During development the proximal polyA sites are frequently used causing mRNAs to have shorter UTRs. [19] Highly proliferative cells have also been shown to have shorter 3’UTRs [20]. Longer UTRs have a greater number of cis-acting regulatory sites and so are likely under greater post-transcriptional control resulting in changes in protein level [6, 21, 22].

Alternate TSSs, splicing and alternate polyA site selection can all result in changes in the protein product produced from an mRNA variant. In addition they can affect sequences within the 5’ and 3’UTRs of an mRNA. The UTRs harbour many of the control elements that dictate when, where and at what rate an mRNA is translated. They also regulate the stability of an mRNA. This is mediated by many trans-acting factors in the form of RNA binding proteins and miRNAs that bind to the UTRs of target mRNAs. These post-transcriptional controls can greatly impact on protein levels in a cell. Recently the translation rate of different mRNA isoforms was compared using RNAseq in a human kidney cell line. It was reported that approximately 30% of mRNA isoforms are differentially loaded with ribosomes suggesting they are translated at a different rate. [23] Recent studies in mouse ESCs demonstrated that different splice variants of *SERCA2* can be differentially targeted by miRNAs in ESCs and embryoid bodies due to differences in UTR sequences [16]. These and other studies highlight the importance of isoform specific UTR sequences in post-transcriptional control of gene expression [24].

We sought to address the impact of alternate UTRs on translation rate of different variants in mouse embryonic stem cells (ESCs) and ESC derived neural precursor cells (NPCs). We have separated mRNAs based on ribosomal load and assessed the relative translation rate of individual variants using RNA seq. We find that there are a significant proportion of variants that show differential translation rates in ESCs and NPCs. We further show that this altered translation rate correlates with processing events that affect 5’ and 3’ UTR sequences with 5’UTR sequences having the greater role. Our data suggests that analysing the translation state of mRNA variants is essential to get a more accurate read out of the protein levels in a cell.

## Results

### Translationally regulated mRNAs on ESC to NPC differentiation

**Fig 1:**
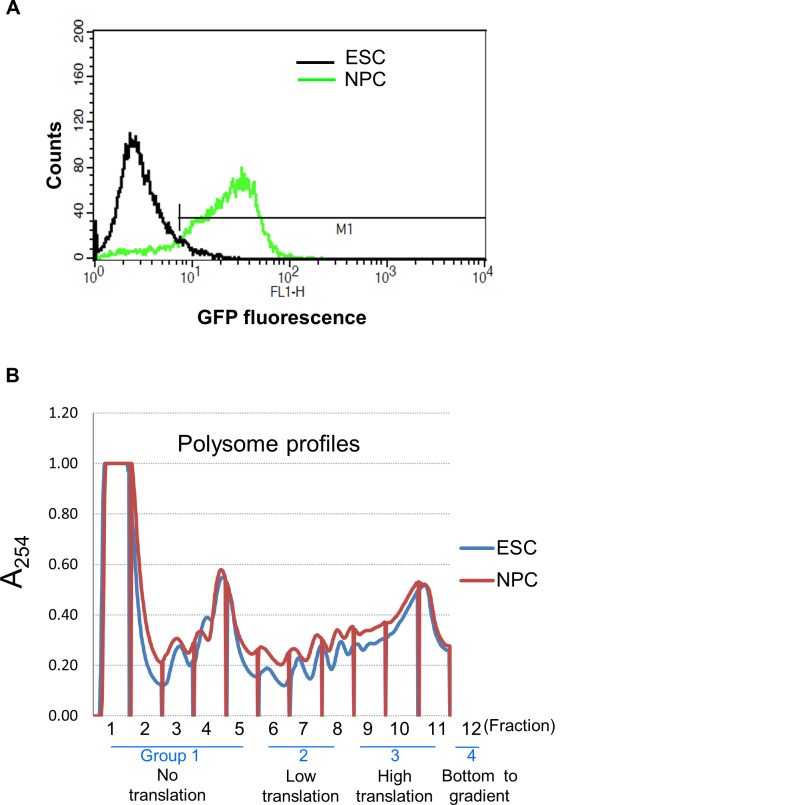
Polysome profiling of ESCs and NPCs. (A) Efficiency of NPC differentiation. Mouse *Sox1-GFP* ESCs were differentiated to NPCs for 6 days. Flow cytometry of the SOX1-GFP signal showing over 80% SOX1-GFP positive cells after differentiation. (B) ESCs and NPCs were subjected to polysome profiling and 12 fractions were collected into four groups. No translation F1-5), Low translation (F6-8), High translation (F9-11) and the bottom of the gradient (F12).

We set out to determine the role of splicing in the regulation of translation rate in ESCs and ESC derived NPCs. ESCs were differentiated into NPCs in a SOX1-GFP reporter line in N2B27 media for six days [25–27]. SOX1 is a marker for NPCs enabling us to confirm efficient differentiation with over 80% of cells expressing SOX1-GFP (Fig 1A). To determine the translation state of RNAs in the two cell types, polysome profiling was performed to separate the differentially translated RNAs across a sucrose gradient. RNAs were pooled together as non-translated, low-translation and high-translation depending on their ribosomal load (Fig 1B). RNAs with a greater ribosomal load sediment in the heavier sucrose fractions and are considered to be more highly translated. RNAs with few or no ribosomes are found in the lighter fractions and are considered to be non- or inefficiently translated. RNAs were also collected from the bottom of the gradient. To determine the translation rate of individual splice variants, we subjected each group of RNAs to RNA sequencing using the ABI SOLiD sequencing platform. Sequences were mapped to the UCSC mm10 reference genome and data was normalised to a pool of four bacterial poly(A) spike-in mRNAs. The location in the polysome profile of a selection of RNAs was validated by qRT-PCR and found to have a good correlation (R^2^-0.7134), suggesting our RNA sequencing gave an accurate read out of the polysome association of individual mRNAs. (S1 Fig)

**Fig 2:**
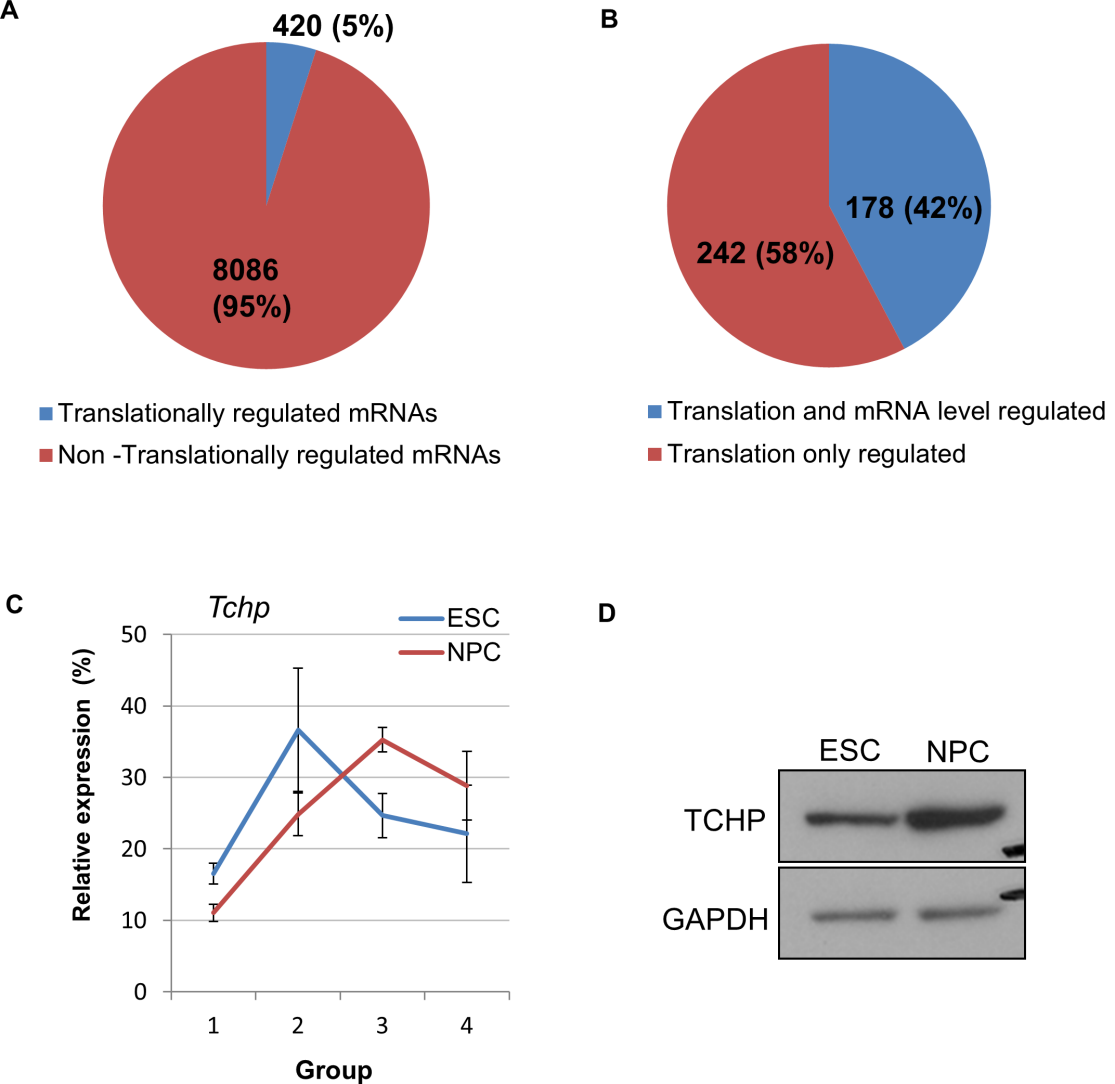
Transcriptional and translational changes upon differentiation of ESCs to NPCs. (A) Pie chart showing the degree of translational shift of mRNAs during ESC to NPC differentiation. 5% of mRNAs display a translation shift greater than 20% upon NPC. (B) Pie chart showing transcriptional changes in the sub-fraction of translationally regulated genes. 58% of these genes are purely regulated translationally and show less than 2 fold changes in total mRNA levels. (C) Real-time PCR analysis of *Tchp* showing increased enrichment in the heavy polysome fractions in NPCs. (D) Western blot of TCHP protein in ESCs and NPCs. GAPDH is shown as a loading control.

A shift in ribosomal load between ESCs and NPCs represents a likely change in translation rate. We saw that 5% of mRNAs showed differential translation rates between ESCs and NPCs with a translation shift of over 20% (Fig 2A). 26% of mRNAs showed a greater than 2 fold change in total RNA levels confirming earlier observations that the transcriptome is dramatically reorganised on ESC differentiation [28]. Interestingly, the majority of translationally regulated RNAs were not co-regulated at the mRNA level. 58% of translationally regulated mRNAs showed less than 2 fold change in RNA levels suggesting they are predominantly translationally regulated (Fig 2B). We used RT-PCR to confirm the polysome association of one translationally regulated mRNA *Tchp*. The increased translation was further indicated from the increased protein levels seen by western blot in NPCs compared to ESCs (Fig 2C and 2D).

### Many splice variants are differentially translated

**Fig 3:**
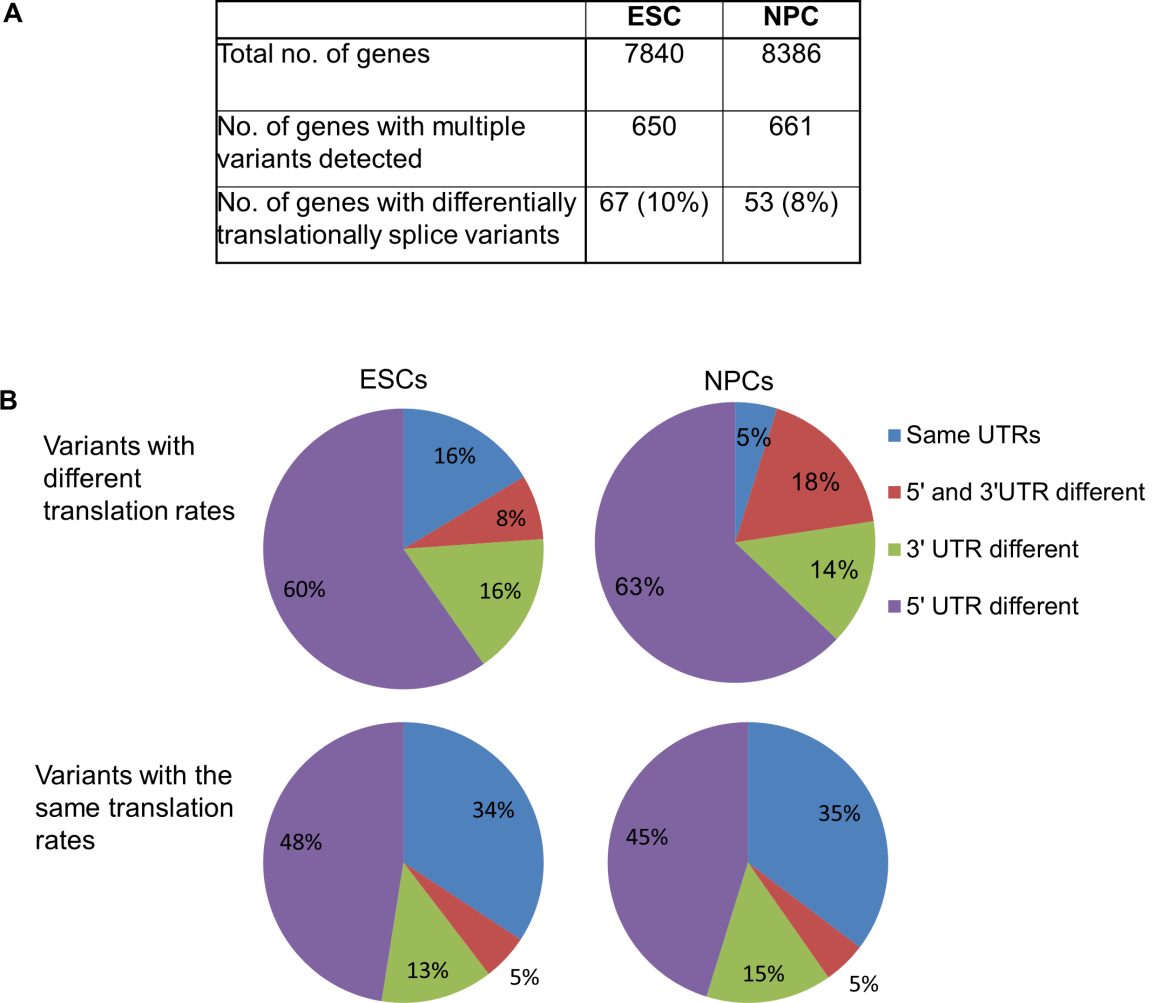
Translational regulation of splice variants in ESCs and NPCs. (A) Analysis of the translation rate of all mRNAs with more than one variant in ESCs and NPCs. (B) Charts showing the correlation between variants with different translation rates and altered UTRs.

We next sought to determine if splice variants displayed translation rate differences within the same cell type. We identified multiple splice variants for 650 genes in ESCs and 661 genes in NPCs (Fig 3A). Of these, 10% and 8% of splice variants were enriched in different polysome pools in ESCs and NPCs respectively (S1 Table). This suggests the splice variants are being translated at different rates within the same cell type. We next assessed the correlation between altered 5’ or 3’ UTRs and different translation rates between the variants. Interestingly, we saw an enrichment for variants with altered UTRs in the differentially translated group of variants compared to variants that show similar rates of translation (Fig 3B). In ESCs, for variants that showed different translation rates, 84% of variants had different UTRs (95% in NPCs). This is in contrast to variants that do not show changes in translation where only 66% of variants displayed altered UTR sequences (65% in NPCs). The majority of variations in both cases were in the 5’UTR with over 60% (63% in NPCs) of translationally regulated splice variants having different 5’UTR sequences. 16% (14% in NPCs) had different 3’UTR sequences and 8% (18% in NPCs) had different 5’ and 3’ UTRs. We analysed the 5’UTRs of mRNA variants that showed differences in translation rate and altered 5’UTRs. We found no significant difference in the GC content of the UTRs however there was an enrichment for longer UTRs in the more translationally repressed variants (S2 Fig). It is likely that the longer sequences contain regions that inhibit ribosome scanning and thus repress translation. There was also a correlation between translation rate and 5’UTR free energy levels such that variants that were more highly translated had a greater free energy level in their 5’UTRs. A higher free energy generally results in less stable RNA structures that could be more conducive to increased ribosomal scanning and thus increased translation (S2 Fig)

Interestingly 16% (5% in NPCs) of variants that showed different translation rates did not have an altered 5’ or 3’ UTR suggesting the regulatory region for translation rate resides in variant regions within the coding sequence for these mRNAs. An example of this is *Ctage5* which has three variants. Variant 1 and 3 have the same UTR sequences but differ only in the inclusion of exons 5 and 6 in variant one. Inclusion of these exons in variant 1 results in a significantly decreased ribosomal load compared with variant 3 suggesting it is being translated at a slower rate. Variant 2 excludes these exons in addition to having a different 3’UTR and its translation is significantly stronger than variant 1 (S3 Fig). Exon 4 and 5 do not have an enrichment of rare codons suggesting the translational repression seen is not due to codon usage. It is likely that exon 5 and 6 contain cis-acting regulatory sequences that might bind to miRNAs or RBPs to repress translation of that variant.

mRNAs with long ORFs may be bound by a higher number of ribosomes which could contribute to the differences in ribosomal load we see between splice variants in one cell type. To see if ORF length was responsible for the altered ribosomal load seen on the different splice variants, we determined the ORF lengths for all variants showing a polysome shift and looked for a correlation with direction of the shift. We saw no strong correlation between longer ORFs and higher ribosomal load (Table 1) (S1 Table). For variants that were bound by more ribosomes less than half had longer open reading frames. This suggests that altered ORF length is not the predominant cause of the altered ribosomal load we see in different splice variants.

**Table 1:** Relationship between ORF length and translation rate.

	ESC	NPC
Genes with multiple variants that show different translation rates correlating with ORF size.	33	20
Genes with multiple variants that show different translation rates **not** correlating with ORF size.	34	33
**TOTAL**	67	53

**Fig 4:**
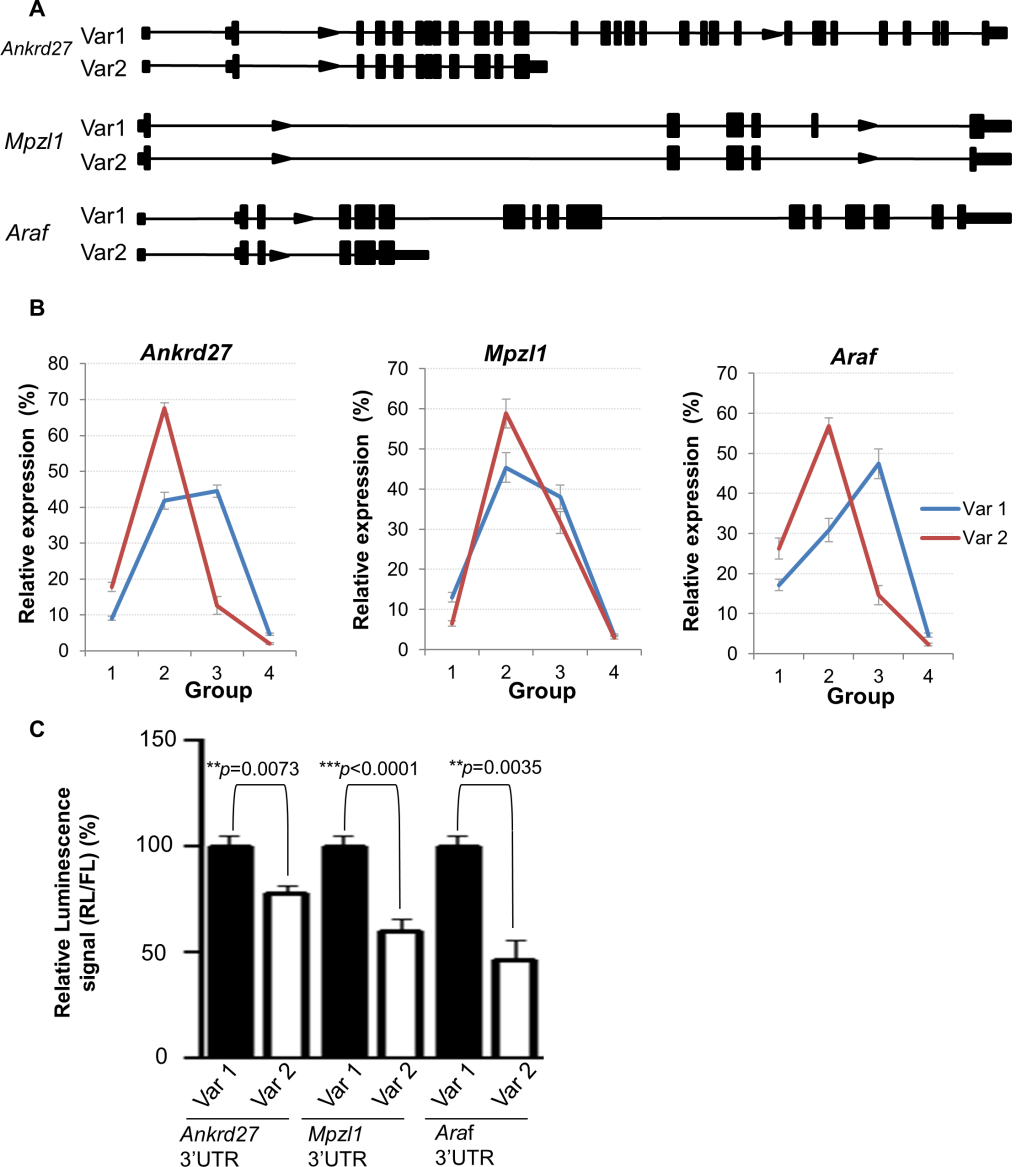
Translational regulation of splice variants with different 3’UTR in ESC. (A) Diagram showing the gene structure for three genes, *Ankrd27*, *Mpzl1* and *Araf*, whose variants are differentially loaded with ribosomes in ESCs. (B) qRT-PCR analysis of the polysome distribution of two variants of three genes in ESCs. (C) Luciferase assays showing the different 3’UTRs of *Ankrd27*, *Mpzl1* and *Araf* variants mediate translational control.

In order to validate the splice variant changes in translation we identified we first performed qRT-PCR with variant specific primers across three biological replicates. We selected three genes that have variant 3’UTRs, *Ankrd27*, *Mpzl1* and *Araf* (Fig 4A). qRT-PCR on the different polysomal groups confirmed that the variants are differentially loaded with ribosomes (Fig 4B). To determine if this difference in translation is mediated through the 3’UTR we cloned the different 3’UTRs downstream of the luciferase reported gene in the psiCHECK-2 vectors. On transfection into ESCs we determined the luciferase levels of each variant. We saw that in all three cases the variant that displayed the lower ribosomal load had the lower luciferase expression levels (Fig 4C). These data strongly suggest that sequences within the UTRs of these genes are responsible for the different translation rates displayed by the variants.

**Fig 5:**
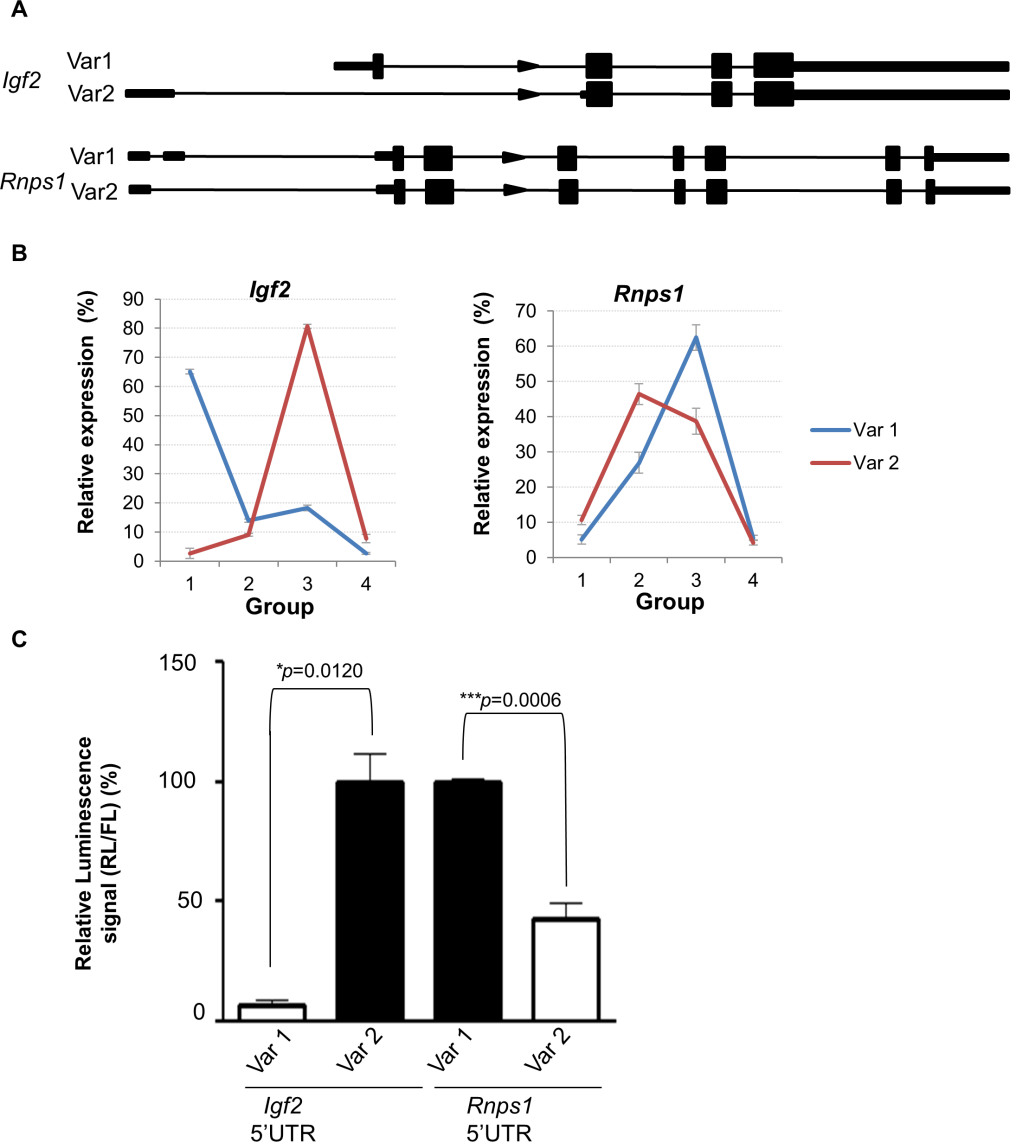
Translational regulation of splice variants with different 5’UTR in ESC. (A) Diagram showing the gene structure for two genes, *Igf2 a*nd *Rnps1*, whose variants are differentially loaded with ribosomes in ESCs. (B) qRT-PCR analysis of the polysomes distribution of two variants of *Igf2* and *Rnps1* in ESCs. (C) Luciferase assays showing the different 5’UTRs of *Igf2* and *Rnps1*variants mediate translational control.

We validated two variants that showed altered 5’UTRs (Fig 5A). qRT-PCR confirmed the differential ribosomal load on polysome fractions (Fig 5B). *Igf2* variant 1 (*Igf2v1*) was enriched in the non-translated fractions while *Igf2* variant 2 (*Igf2v2*) was enriched in the highly translated fractions. For *Rnps1*, variant 1 (*Rnps1v1*) was in the heavy polysome fractions while variant 2 (*Rnps1v2*) was in the low translation fractions. When the 5’UTRs of these mRNAs were cloned upstream of the luciferase reporter gene they caused a decrease in luciferase activity correlating with the changes in ribosomal load seen by PCR (Fig 5C). These data suggest that the *Igf2v2* 5’UTR promotes translation compared to the *Igf2v1* 5’UTR, and the *Rnps1v2* 5’UTR promotes a reduction in translation compared to variant 1. The exclusion of exon 2 in *Rnps1v2* results in the creation of an 81 nucleotide long upstream open reading frame (uORF) that may function to repress translation of that variant. Interestingly, the ORF for *Rnps1* variants 1 and 2 are identical, so expression of different isoforms in different cell types has the potential to influence protein production through the presence of the uORF. *Rnps1* forms part of a pre- and post-splicing RNA bound complex that plays a role in splicing, nuclear-cytoplasmic shuttling and RNA stability [29–31]. These data suggest that the alternative UTR sequences seen in different mRNA isoforms can greatly impact the translation rate of mRNA variants.

Variant specific translation likely plays an important role in ESC differentiation. We detect 31 genes that have at least one variant that is selectively translationally regulated on differentiation of ESCs to NPCs (S2 Table). These variants are regulated independently from the other present variants of that gene. One example is *Rnps1* (discussed above) where variant1 is highly translated in ESCs and NPCs. Variant 2 is more translationally repressed in ESCs and its polysome association is increased in NPCs indicative of increased translation (S4 Fig).

### Non-coding mRNAs are enriched on polysomes

**Fig 6:**
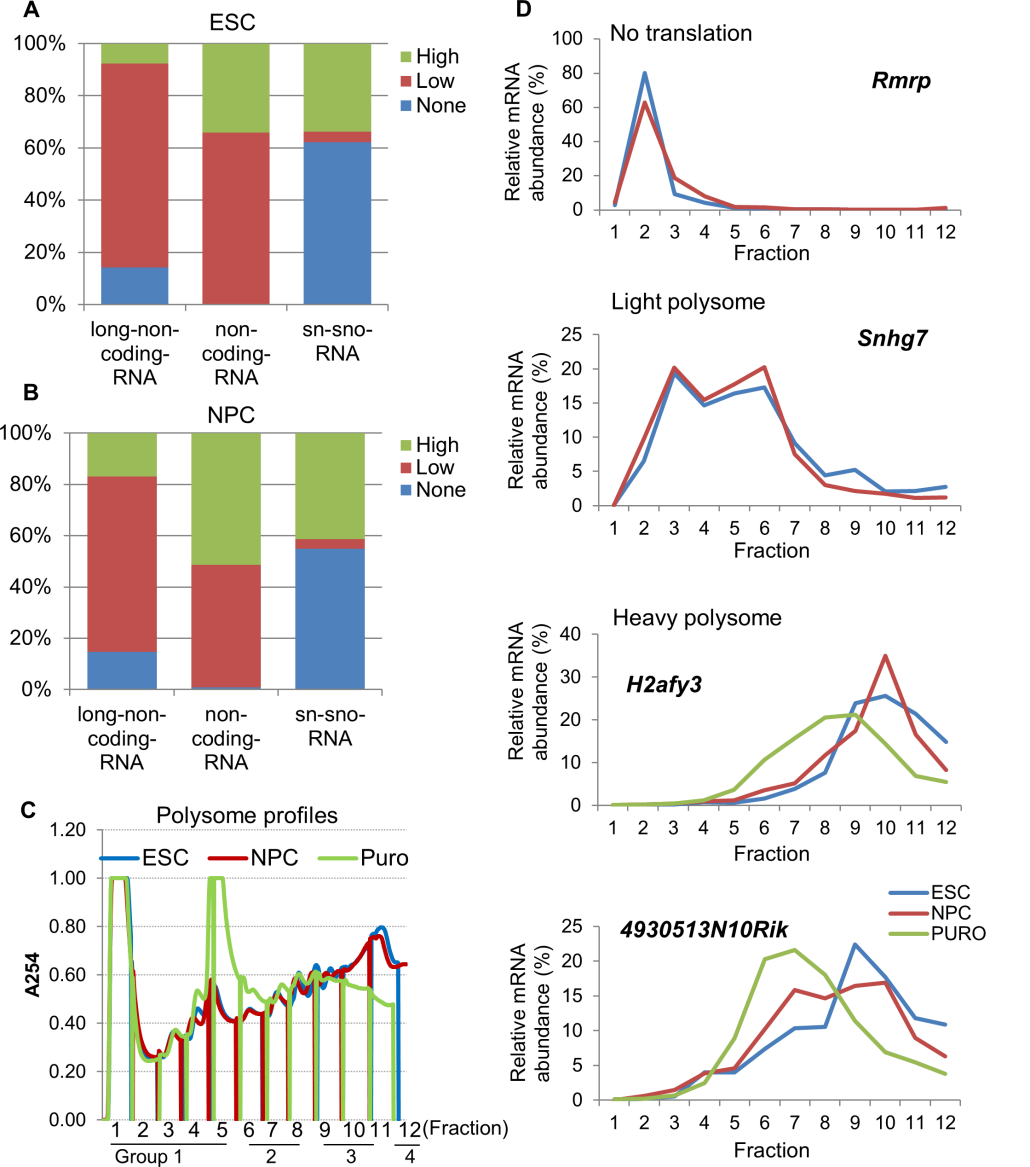
Polysomal association of ncRNA in ESC and NPC. (A,B) Analysis of the distribution of annotated non-coding RNAs in the different polysome fractions of ESCs and NPCs. (C) Polysome profiles of ESCs, NPCs and puromycin treated ESCs (PURO) to selectively disrupt polysomes. (D) qRT-PCR of representative non-coding RNAs showing their distribution in polysome gradients from ESCs and NPCs with and without the addition of puromycin.

Recently it has been demonstrated that many non-coding mRNAs are present on polysomes. A significant percentage of annotated non-coding mRNAs have ribosomal footprints following ribosomal footprint analysis suggesting they are engaged by the translating ribosome.[32] More recent studies have confirmed that a significant percent of non-coding mRNAs are on the polysomes.[33] Based on this, we determined the polysome association of annotated non-coding mRNAs in our datasets. We identified over 260 GenBank annotated non-coding RNAs in ESCs (280 in NPCs) that include long non-coding RNAs (lnc-RNAs), non-coding RNAs (ncRNAs), small nuclear and small nucleolar RNAs. The lnc-RNAs include predominantly large intergeneic non-coding RNAs (lincRNAs) while the non-coding RNAs include predominantly pseudogenes. In agreement with previous studies we see a large proportion of these non-coding RNAs associated with the light and heavy polysome fractions (Fig 6A and 6B). Over 70% of lnc-RNAs were associated with the low or high translation fractions in both ESCs and NPCs while over 90% of non-coding RNAs were associated with polysomes. We also saw a significant percentage of sn- and sno- RNAs on the polysomes. qRT-PCR validation of a selection of non-coding RNAs showed that *Rmrp* was enriched in the non-translated fractions. *Rmrp* is a component of the ubiquitously expressed RNA-processing endoribonuclease [34]. *Snhg7*, a small nucleolar RNA host gene was enriched in the light polysome fractions and *H2afy3*, a non-coding pseudogene, and *4930513N10Rik* were enriched in the heavy polysome fractions. To confirm that these RNAs were really associated with polysomes and not other heavy RNP particles we treated cells with puromycin to selectively breakdown polysomes (Fig 6C and 6D). *H2afy3* and *4930513N10Rik* were seen to shift from the heavy fractions to the lighter fractions in the presence of puromycin suggesting they are associated with polysomes. These data therefore confirm previous observations that many non-coding RNAs are enriched on polysomes.

## Discussion

We have performed RNA sequencing analysis on mRNA isolated from different fractions of a polysome gradient in order to analyse the translation rate of individual mRNA variants. We find that many mRNAs are translationally regulated during differentiation of ESCs to NPCs. We have validated that a shift in ribosomal load has a consequence for protein production with *Tchp*. *Tchp* is translationally upregulated in NPCs resulting in an increase in protein levels. TCHP was originally described as a keratin filament binding protein and plays a role in ciliogenesis and centrosomal function [35, 36]. TCHP has no known role in the nervous system but given its translational upregulation it may be a regulator of early neural differentiation. We identified 31 genes expressing multiple variants that are translationally regulated in a variant specific manner on NPC differentiation. In these cases multiple variants are detected but only one is under translational control on differentiation. In a number of cases there was a switch in the variant that was highly translated so that while the absolute levels of the RNA variants may be the same, their different translation rates will result in the protein products being present at different levels in ESCs and NPCs. Interestingly the role of the majority of these variants is not known so it remains to be determined how this variant specific translation impacts on neural differentiation.

The method of RNAseq analysis of polysomal fractions is different from the alternative approach of ribosomal footprinting which provides information on the mRNA sequence physically bound by ribosomes and is used to infer translation rate [32, 37]. Despite the high resolution of the method the footprint sequences do not provide information on UTRs. In addition, currently the technique is not able to distinguish between different splice variants. Polysome profiling coupled to RNAseq provides information on variant specific ribosomal load and enables the analysis of corresponding UTR sequences. Using polysome profiling coupled with RNAseq, we find that in ESCs ten percent of RNA isoforms display different translation rates. This correlates with differences in the UTR sequences which likely drive the altered translation rate. This suggests that the relative protein levels of the two isoforms will not correlate with their mRNA levels.

Alternate UTRs can arise through a number of different mechanisms including alternate TSS, alternative splicing and alternative polyA site selection. Our datasets were mapped to the RefSeq genome which has annotations for promoter start sites and known alternative splicing events. As such, our studies have focused only on variants arising from alternative transcription start site selection and alternative splicing. Our analysis does not take into account any APA that may be occurring and it is possible that the variants identified in our studies may be further regulated by APA in different cell types. We find that in ESCs 10% of mRNAs with multiple variants display different translation rates for each variant inferred from their ribosomal load. This strongly correlates with the variants having different 5’ and 3’UTR sequences. This represents over 67 genes with different detectable isoforms in ESCs. This demonstrates the importance of analysing the translation rates of RNA variants when studying gene expression patterns. We confirmed the differential ribosomal load of five variants by PCR and performed luciferase assays to confirm that the regulation was mediated through sequences in the UTRs of these mRNAs. We tested two variants that had altered 5’UTRs and three that had altered 3’UTRs. In all cases the UTR sequence was shown to regulate luciferase protein levels in a similar way to that predicted by the polysome profiling data. Where the variant had a decreased ribosomal load following polysome profiling, the UTR promoted a decrease in luciferase activity suggesting a role in translational repression. It is likely that the UTRs that are associated with a decreased ribosomal load have cis-acting sequences that confer translational repression. These could take the form of binding sites for miRNA or RNA binding proteins. Alternate 5’UTRs could also contain uORFs which can function to repress translation [38, 39]. The variants that do not have these sequences are likely not targeted for repression. These different UTR sequences have likely evolved to confer additional levels of regulation on specific RNA variants and have the potential to precisely regulate their translation both spatially and temporally.

Analysis of the ORF size of the translationally regulated variants suggested that for most variants the differences in ribosomal load were not a consequence of altered ORF size. There were a minority of variants that had significantly longer ORFs that correlated with increased ribosomal load and in these cases the ORF length is likely the cause. For variants that did show a correlation between ORF length and ribosomal load the difference in size was not large enough (less than 25% larger) to result in a shift in the polysome fractions in most cases (Table 1). Interestingly we identified a selection of splice variants that had different translation rates but no change in the UTRs. While some of these candidates will have a decreased ribosomal load due to a decreased ORF length it is likely that the remaining transcripts contain cis-acting regulatory sequences within their ORF. miRNAs have been shown to target the ORF of mRNAs [40, 41] so it is possible that the changes in ORF seen in these variants results in altered translational control in addition to altered protein function. Exon 4 and 5 in *Ctage5* variant 1 are associated with a decreased ribosomal load compared to variant 3 that skips these exons. While there was no enrichment for rare codons in these exons it is possible that these regions could bind to trans-acting factors or that the sequence itself is inhibiting translation. It will be interesting to determine if these exons can function to regulate translation from within the UTR or if they have to be in the ORF to function.

We assessed the ribosomal load of annotated non-coding RNAs and found that a significant number of long non-coding RNAs (lncRNAs) and non-coding RNAs were associated with the ribosomes. The lnc-RNAs include predominantly long intergenic non-coding RNAs (lincRNAs) while the non-coding RNAs include predominantly pseudogenes. The presence of the pseudogenes on the polysomes is in agreement with recent reports that analysed ribosomal footprinting data from a number of different species. [42] Recent studies have demonstrated the presence of many non-coding RNAs on polysomes. Our studies confirm that many non-coding RNAs are in the polysome fraction but we cannot distinguish between RNAs that are actively being translated by the ribosome and those that may be binding the ribosome or other mRNA molecules that are being translated. It is possible that many non-coding RNAs could be associating with the ribosome in a regulatory capacity and so while associated with the ribosome would show no ribosomal footprint. It has been suggested that non-coding RNAs could be being translated to give short peptides but additional studies are needed to elaborate on this idea [32, 42, 43]. Our data confirms the presence of non-coding RNAs on the ribosome and further investigation is needed to determine why they are there.

Taken together our data illustrates the importance of addressing the translation rate of individual mRNA variants. We demonstrate that different mRNA variants can have very different translation rates. This confirms and expands on previous reports from human cells and demonstrates variant specific translation rates in ESCs and NPCs. Additional work is needed to determine the mechanisms by which these mRNAs are regulated and their significance for ESC self-renewal and pluripotency.

## Experimental Procedures

### Cell Culture

Sox1-GFP ESCs were a gift from Dr Austin Smith [26, 27]. ESCs were cultured on 0.1% gelatin in DMEM (GIBCO) supplemented with 15% fetal bovine serum (FBS) (GIBCO), 0.2 mM β-mercaptoethanol, 2 mM L-glutamine (GIBCO), 13 MEM nonessential amino acids (GIBCO), and LIF. For NPC differentiation Sox1-GFP ESCs were plated at low density and grown in N2B27 media for 6 days NPC as described [26]. The efficiency of NPC differentiation (>80%) compared to ESC was confirmed by detection of GFP fluorescence signal using flow cytometry.

### Polysome Fractionation and RNA extraction

Polysomes were isolated as described previously [5] and equal OD units were loaded onto 10%–50% linear sucrose gradients and centrifuged at 36,000 rpm for 1 hour and 45 min at 8°C in a SW41 rotor (Beckman Coulter). Twelve fractions were collected from the top of the gradient using a piston gradient fractionator (BioComp Instruments). The absorbance at 254 nm was measured with a UV-M II monitor (Bio-Rad).

Following fractionation, 110 μL of 10% SDS and 12 μL of proteinase K (20 mg/mL; Invitrogen) were added to each fraction and incubated with shaking at 1000 rpm for 30 min at 42°C to digest protein debris and to inactivate nucleases. Fractions of 1–5, 6–8, 9–11, 12 were pooled to give groups 1–4, which we classify as no translation, low translation, high translation and mRNAs present at the bottom of sucrose gradient respectively. Spike-in RNAs from the GeneChip Eurkaryotic Poly-A RNA control kit (Affymetrix) were added to each group of fractions (20 μl, 12 μl, 12 μl and 4 μl of 200-fold diluted spike-in RNA into group 1–4 respectively). Polysomal RNA was extracted by phenol chloroform isoamyl alcohol (PCI) and then chloroform isoamyl, further followed by subsequent purifications using sodium chloride (NaCl), lithium chloride (LiCl) and sodium acetate (NaOAC). RNA quality was assessed on the Agilent Bioanalyzer using RNA6000 Nano kit.

### Library preparation and sequencing

The twelve polysomal fractions from ESCs and NPCs were pooled and placed into groups that were indicative of their translation rates. Fractions 1–5 were pooled as non-translated mRNAs (non). Fractions 6–8 were pooled into the “low” group containing mRNAs translated at a low rate. Fractions 9–11 were pooled into the “high” group and contained the highly-translated mRNAs. Fraction 12 was collected as the “unknown” group consisting of the cell debris and very large RNPs. Total unfractionated RNA was kept as a control. Each of the pooled groups were subjected to Library preparation and SOLID^TM^ sequencing. The reads obtained were single-end reads and were 75bp in length.

### Alignment, normalization and scaling of the reads

The flowchart of the data analysis is given in S5 Fig. Briefly, the reads for each of the four groups and the total unfractionated RNA for both the ESC and NPC were aligned to the *Mus musculus* genome (mm10) using LifeScope^TM^. The bam files comprising the alignments were then imported into Partek Genomics Suite. The allocation of reads was done through Partek using the “Expectation Maximisation” algorithm. This was followed by RPKM normalisation. The normalized reads were scaled using scaling factors calculated from the polyA spike-in RNAs. The relative proportion of the spike-in RNAs in each polysome group was determined and used to calculate the scaling factor taking into consideration the original sample volume (S6 Fig). The scaled normalised reads were converted into percentage of reads in each pool where the sum of the pools was 100%. Transcripts with an unscaled RPKM value > = 0.5 in each of the four groups and an RPKM value of > = 5 in at least one of the four groups were analysed. The data discussed in this publication has been deposited in NCBI's Gene Expression Omnibus (Edgar et al., 2002) and is accessible through GEO Series accession number GSE73467

### Analysis of translation rates

To identify mRNAs translationally regulated between ESCs and NPCs transcripts were analysed if they had an unscaled RPKM value > = 0.5 in all polysome fractions in ESCs and NPCs and an RPKM value of > = 5 in any one of the eight groups. mRNAs showing a shift in percentage in any fraction of > = 20 between ESCs and NPCs were considered to be translationally regulated. Total mRNA level was determined by taking the sum total of scaled data in the four groups. A fold change cut-off of 2 fold was applied to obtain transcripts that had different mRNA levels.

To analyse translation rates of splice variants in ESCs or NPCs all genes with multiple detected variants were analysed. The scaled percentages of the variants were compared within the same cell type. Variants predicted to be non-coding were removed to ensure only translational control events were identified. Genes with variants showing a percentage change of > = 20 in any group were classified as having “Translationally Regulated Splice Variants”. Genes with variants not showing a percentage shift > = 20 in any of the groups were classified as genes having “Non Translationally Regulated Splice Variants”

### Correlation of ORF and UTR length with translation rate

The ORFs and 5' and 3' UTR sequences were retrieved from the UCSC table browser (*Mus musculus* genome mm10). The ORF lengths of the translationally regulated splice variants were compared. ORF lengths within 10nt of each other were considered to be the same. These were correlated with the translation rates of the variants. The lengths of the UTRs of different variants were calculated and compared to the translation rate of each variant.

### 5’UTR correlation studies

For each gene in the TR sets, the translation efficiency of each variant was correlated with the GC content, Free Energy and length of the 5’UTR. Genes were determined to have a direct correlation when increased translation correlated with increased GC content, free energy or length of the 5’UTRs. Genes were classified as having a direct correlation, opposite correlation or no correlation. For genes where no clear conclusion could be drawn due to the presence of equal numbers of variants showing different trends, they were labelled as undecided.

### Non-coding RNA polysomal enrichment analysis

The non-coding RNA transcripts annotated as “NR” were classified according to the pool in which they were most represented. All analysed non-coding RNAs were enriched in one of the NONE, LOW or HIGH translation pools.

### Semi-quantitative Real-Time Polymerase Chain Reaction

cDNA was synthesized using a reverse transcription kit (Super-Script III, Invitrogen) according to the manufacturer’s instruction. For fractionated RNA, the same volume of RNA was used, and for total RNA analysis, the same quantity of RNA was used. SYBR Green was used with transcript-specific primers for qRT–PCR on an ABI PRISM 7900 sequence detection system. For polysome fractions, CT values were normalized to spike-in control RNAs dap and thr. Primers were either purchased from Integrated DNA Technologies (IDT) or designed. Expression levels detected by qPCR for each group are presented as percentage of RNA in each fraction relative to the total values of 4 groups.

### Luciferase reporter assay

The UTR of candidate mRNAs were cloned into the psiCHECK-2 vector. ESCs were transfected using FuGENE HD (Promega) according to manufacturer’s instructions. Thirty hours after transfection, cells were lysed and Renilla (RL) and firefly (FL) luciferase activities were determined using the Dual-Luciferase Reporter Assay system (Promega). For data analyses, all RL signals were normalized by the non-targeted control FL readings.

### Western blot analysis

20ug of protein extract was separated on a NuPAGE 4–12% Bis-Tris Gel and transferred to PVDF membrane. TCHP (ARP60515_P050, Aviva Systems Biology), and GAPDH (Abcam Cat No.ab-9484) antibodies were used at 1:1000 dilution.

### Statistical Analysis

Luciferase activities in ES cells transfected with different psiCHECK-2 plasmids were compared using the paired Student’s t-test. For qRT-PCR and luciferase reporter assays, the standard error of the mean is shown. * P<0.05

## Supporting Information

S1 Fig Correlation between RNAseq and qRT-PCR results. The sequencing data shows a high level of correlation with qRT-PCR.
(TIF)

S2 Fig Correlation between translation rate of variants and 5’UTR properties. Analysis of the 5’UTRs of variants that showed different translation rates and different 5’UTRs. 5’UTRs were assessed for their level of GC content, free energy and length and compared between the different variants.
(TIF)

S3 Fig ORF mediated translational control of splice variants. (A) schematic showing the different variants of *Ctage5*. (B) Enrichment of the three *Ctage5* splice variants in different polysome fractions.
(TIF)

S4 Fig Variant specific translation on NPC differentiation. (A) schematic showing the different variants of *Rnsp1*. (B) Real-time PCR showing the enrichment of the two *Rnsp1* splice variants in different polysome fractions. Variant 2 is translationally more repressed in ESCs but is translationally activated in NPCs. Variant 1 is highly translated in both ESCs and NPCs.
(TIF)

S5 Fig Mapping statistics of ESC and NPC RNA seq data. (TIF)

S6 Fig Calculation of scaling factors for normalization. (TIF)

S1 Table Translationally regulated splice variants in ESC and NPC. Table showing the different ribosomal loads of variants within ESCs and NPCs. The data is presented as percentage of transcript present in each group. ORF lengths are also shown.
(XLSX)

S2 Table Variant specific translational regulation on ESC to NPC differentiation. Table showing the expression levels of different mRNA variants in different polysome fractions on differentiation of ESCs to NPCs. The scaled data is shown along with the percentage of transcript present in each group. All expressed variants are shown with at least one variant showing a change in translation rate in a variant specific manner.
(XLSX)

## Abbreviations

ESC: Embryonic Stem Cell
NPC: Neural Precursor Cell
