# Supplementary figures and images for: Embryonic Stem Cells Exhibit mRNA Isoform Specific Translational Regulation
Source: PLoS One. 2016 Jan 22;11(1):e0143235. doi: 10.1371/journal.pone.0143235 (PMC4723142; doi:10.1371/journal.pone.0143235)

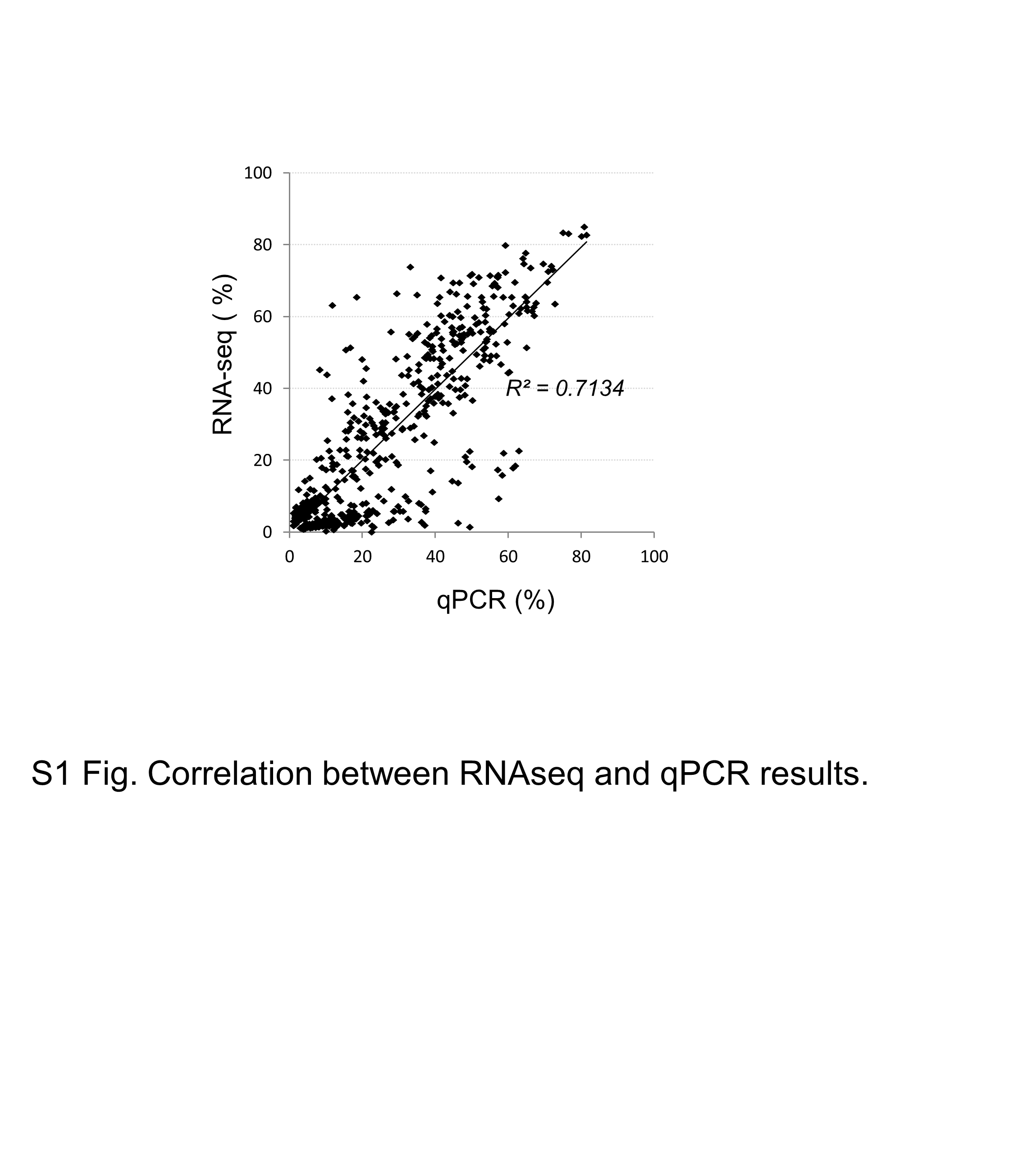

Supplement: S1 Fig — The sequencing data shows a high level of correlation with qRT-PCR. (TIF) [file pone.0143235.s001.TIF]

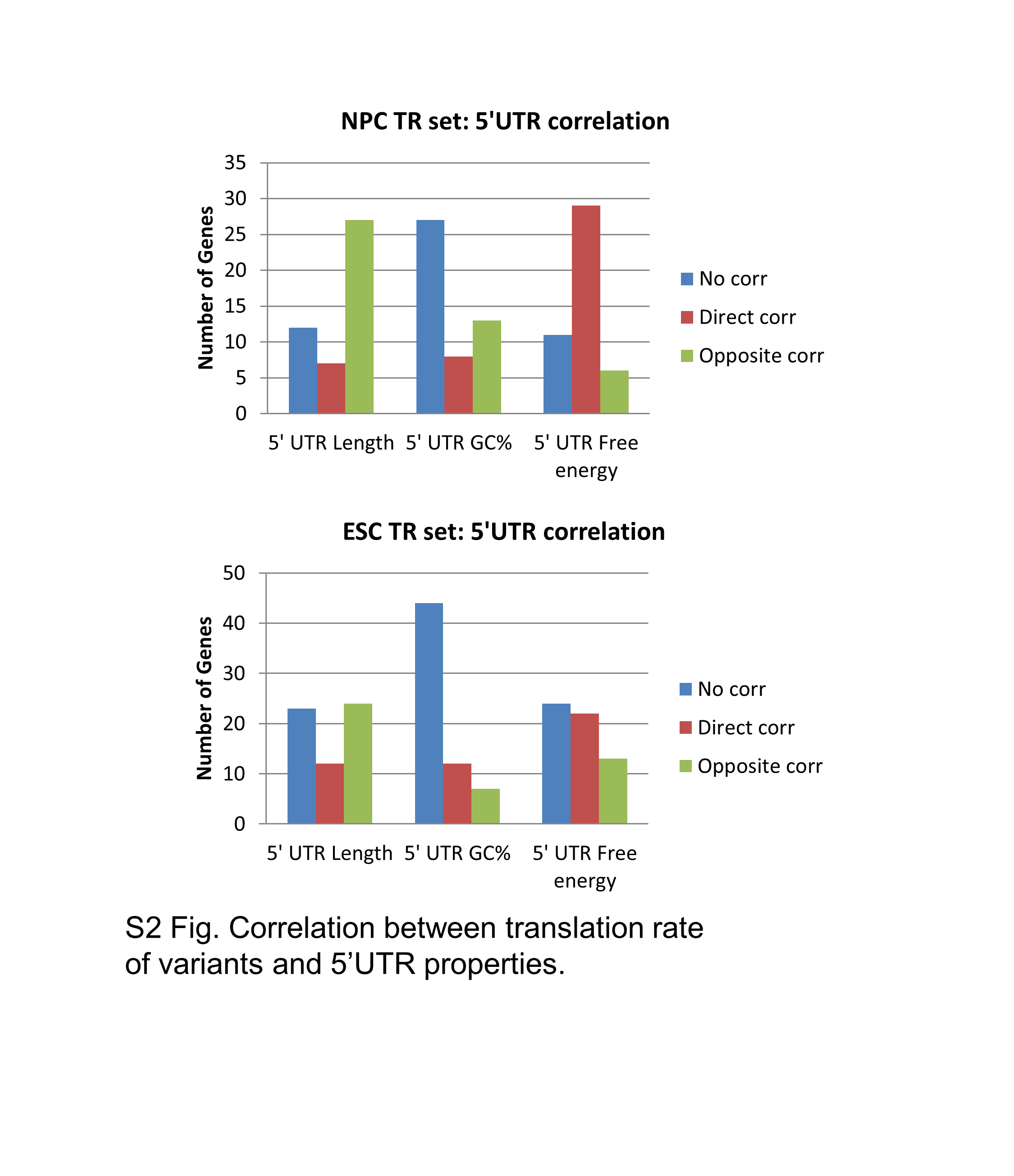

Supplement: S2 Fig — Analysis of the 5’UTRs of variants that showed different translation rates and different 5’UTRs. 5’UTRs were assessed for their level of GC content, free energy and length and compared between the different variants. (TIF) [file pone.0143235.s002.TIF]

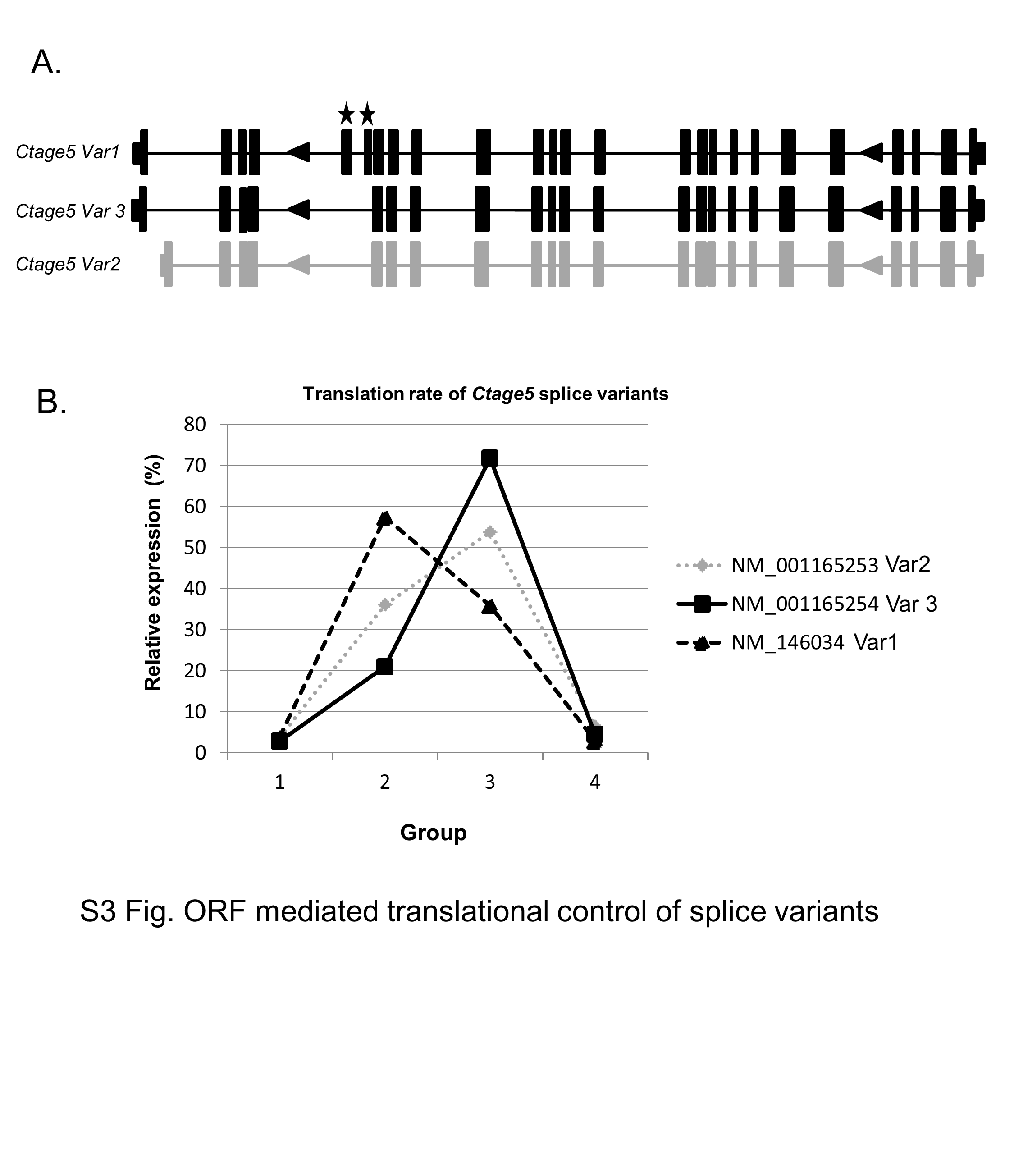

Supplement: S3 Fig — (A) schematic showing the different variants of Ctage5. (B) Enrichment of the three Ctage5 splice variants in different polysome fractions. (TIF) [file pone.0143235.s003.TIF]

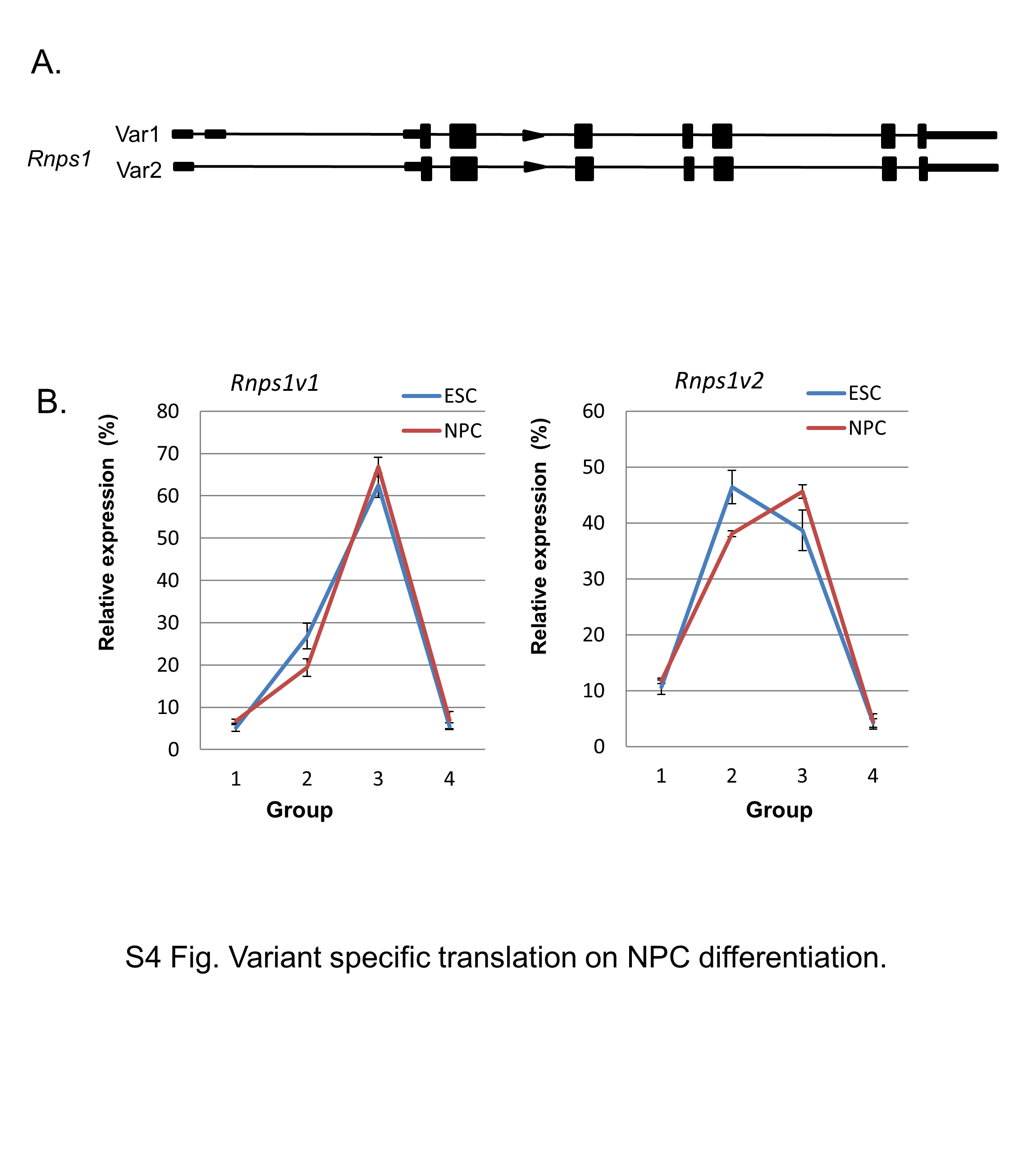

Supplement: S4 Fig — (A) schematic showing the different variants of Rnsp1. (B) Real-time PCR showing the enrichment of the two Rnsp1 splice variants in different polysome fractions. Variant 2 is translationally more repressed in ESCs but is translationally activated in NPCs. Variant 1 is highly translated in both ESCs and NPCs. (TIF) [file pone.0143235.s004.TIF]

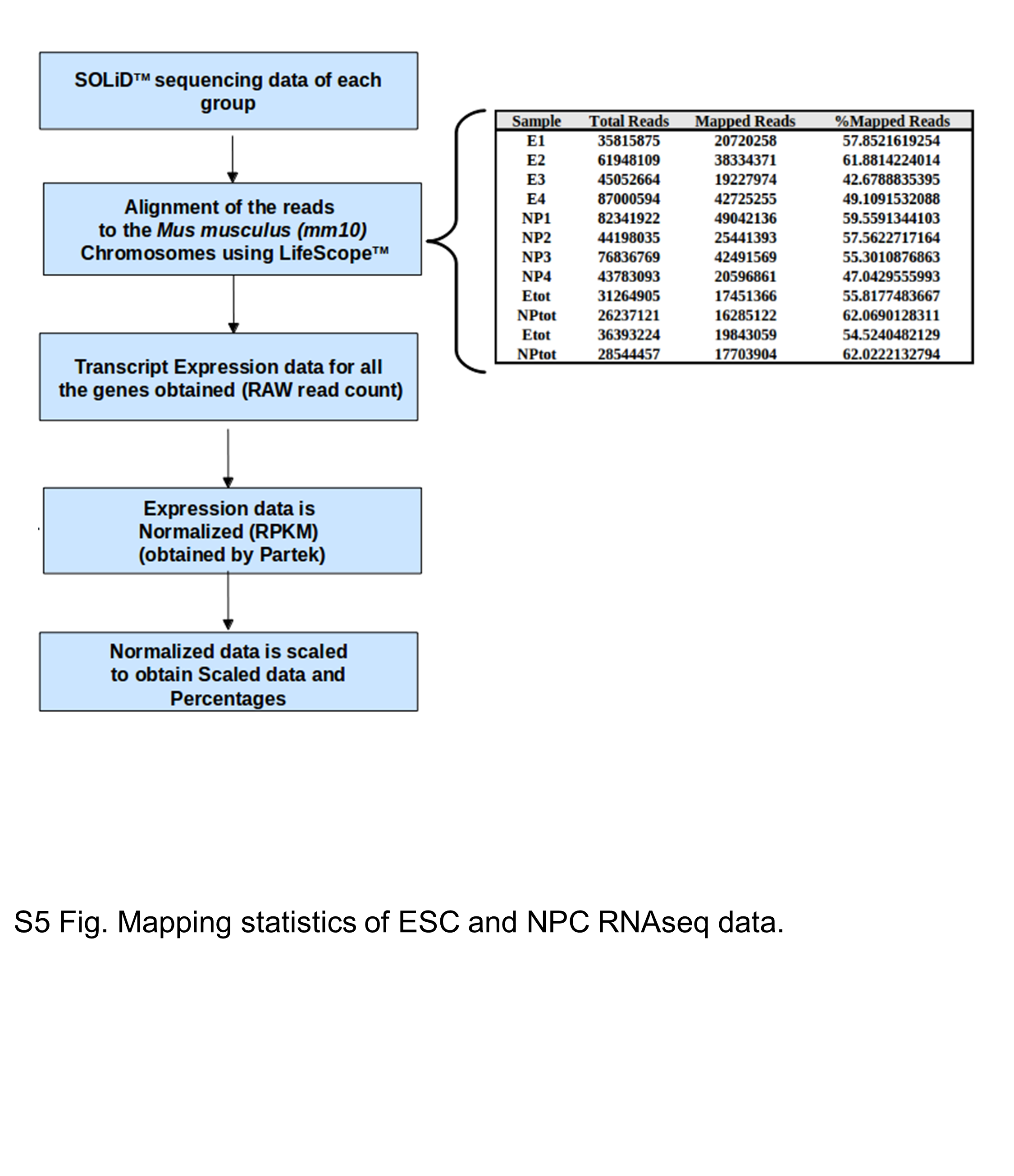

Supplement: S5 Fig — (TIF) [file pone.0143235.s005.TIF]

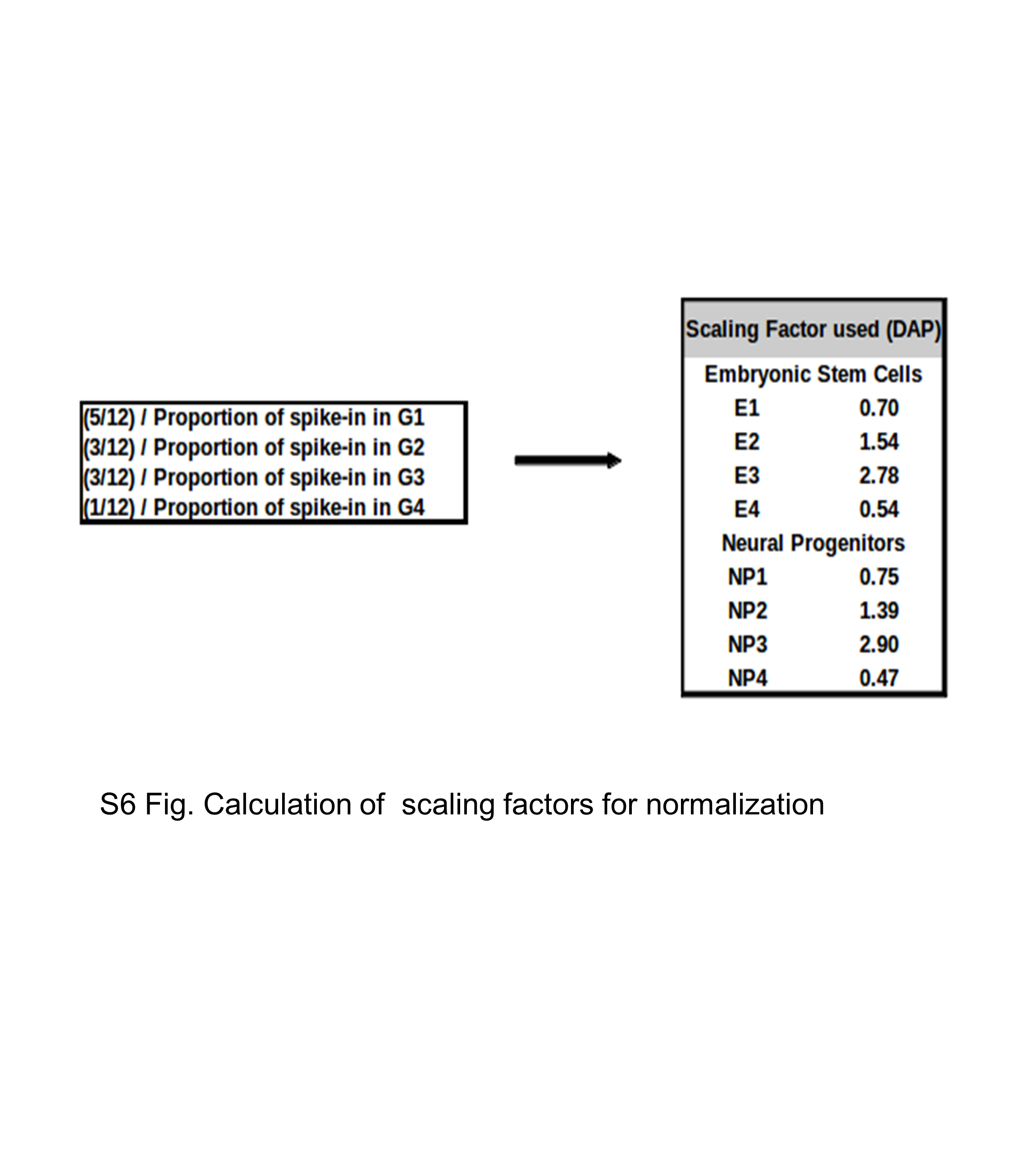

Supplement: S6 Fig — (TIF) [file pone.0143235.s006.TIF]
